# Joint encoding of stimulus and decision in monkey primary visual cortex

**DOI:** 10.1093/cercor/bhad420

**Published:** 2023-11-11

**Authors:** Yang Yiling, Johanna Klon-Lipok, Wolf Singer

**Affiliations:** Ernst Strüngmann Institute (ESI) for Neuroscience in Cooperation with Max Planck Society, Deutschordenstraße 46, 60528 Frankfurt am Main, Germany; Max Planck Institute for Brain Research, Max-von-Laue-Str. 4, 60438 Frankfurt am Main, Germany; Ernst Strüngmann Institute (ESI) for Neuroscience in Cooperation with Max Planck Society, Deutschordenstraße 46, 60528 Frankfurt am Main, Germany; Max Planck Institute for Brain Research, Max-von-Laue-Str. 4, 60438 Frankfurt am Main, Germany; Frankfurt Institute for Advanced Studies, Ruth-Moufang-Str. 1, 60438 Frankfurt am Main, Germany

**Keywords:** choice, dynamics, high-dimensional coding, mixed selectivity, V1

## Abstract

We investigated whether neurons in monkey primary visual cortex (V1) exhibit mixed selectivity for sensory input and behavioral choice. Parallel multisite spiking activity was recorded from area V1 of awake monkeys performing a delayed match-to-sample task. The monkeys had to make a forced choice decision of whether the test stimulus matched the preceding sample stimulus. The population responses evoked by the test stimulus contained information about both the identity of the stimulus and with some delay but before the onset of the motor response the forthcoming choice. The results of subspace identification analysis indicate that stimulus-specific and decision-related information coexists in separate subspaces of the high-dimensional population activity, and latency considerations suggest that the decision-related information is conveyed by top-down projections.

## Introduction

The primary visual cortex (V1) receives not only retinal input but also massive recurrent connections from other cortical areas (van den Heuvel and Sporns 2011; Markov et al. 2013) and ascending projections from modulatory thalamic and brain stem systems (Bear and Singer 1986; Munk et al. 1996). Accordingly, the activity of V1 neurons does not solely depend on visual stimuli (Pinto et al. 2019; Steinmetz et al. 2019; Zatka et al. 2021). However, it is unclear to which extent these non-retinal projections just exert a global modulation of excitability or convey specific information. Evidence from higher cortical areas such as the prefrontal and motor cortex indicates that cortical neurons can have mixed selectivity and as a population jointly encode in parallel different aspects of a task (Churchland et al. 2012; Mante et al. 2013; Rigotti et al. 2013; Elsayed et al. 2016; Parthasarathy et al. 2017; Aoi et al. 2020; Perich et al. 2020). This capacity to generate multidimensional codes is attributed to complex, nonlinear interactions within cortical networks that provide the high-dimensional dynamic space required for such representations (Singer and Lazar 2016; Singer 2021). The intrinsic connectivity of V1 and its embedding in the cortical connectome share numerous similarities with the cortical areas for which multidimensional coding has been demonstrated. V1 also exhibits the required nonlinear, high-dimensional dynamics due to the abundance of recurrent interactions (Singer and Lazar 2016; Singer 2021). Therefore, we hypothesized that V1 might also exhibit mixed selectivity and be able to represent in population responses not only information about visual stimuli but also specific information processed in other cortical areas. To test this conjecture we examined whether neuron populations in V1 could jointly encode both sensory input and information about perceptual decisions or behavioral choices.

To this end, we performed parallel multisite electrophysiological recordings in V1 of awake monkeys engaged in a forced choice, delayed match-to-sample (DMS) task. The task required to judge whether 2 sequentially presented stimuli, the sample and the delayed test stimulus, are the same or different, and to communicate the decision with a 2-way lever response. Applying advanced decoding methods, we examined whether the population responses to the test stimulus contained information of both the visual stimulus and the forthcoming decision.

## Materials and methods

### Behavioral task

Results presented here were obtained from 2 adult rhesus monkeys (*Macacca mulatta*: monkey 1: male, 11 kg, 12 years old; monkey 2: female, 9 kg, 17 years old). All experimental procedures were in compliance with the German and European regulations on laboratory animal protection and welfare and were approved by the local authority (Regierungspräsidium Darmstadt). The monkey (applicable to both monkeys) was seated 60 cm in front of a screen (Samsung SyncMaster 2233RZ; 120 Hz refresh rate) inside a dark booth. The monkey initiated a trial by fixating at a white fixation dot displayed at the centre of the screen and had to maintain fixation on the fixation dot until the trial ended. The eye position was monitored with the EyeLink tracker (SR Research Ltd., Ottawa, Ontario, Canada).

In the DMS task, 2 stimuli were presented sequentially, and the monkey had to report whether the 2 stimuli were the same (match) or different (nonmatch). A trial started with a fixation period of 500 ms, during which the screen was blank. Then the first stimulus (sample) was presented for 500 ms, followed by a delay period of 1500 ms after which the second stimulus (test) was presented. The test disappeared once the monkey responded or kept on for maximally 500 ms in case of delayed responses. The monkey had to respond by moving a 2-way mechanical lever: forward in match trials and backwards in nonmatch trials. The number of match and nonmatch trials was pseudo-randomized to be equal. A correct response was rewarded with a drop of water or juice. If the monkey broke fixation (1.2° around the fixation point), or moved the lever before target onset, the trial was aborted.

### Stimulus design

The stimuli were images of single isolated objects with transparent background (Brodeur et al. 2010; Brodeur et al. 2014). The visible region of the image was normalized to equal pixel intensity (0.5) and root-mean-square contrast (0.275), and covered the classical receptive fields of the recorded multi-units. The image size was 160 × 160 pixels (4.4° visual angle) for Monkey 1, and 250 × 250 pixels (6.9° visual angle) for Monkey 2. The images were shown at 50% transparency (alpha = 0.5) to reduce the influence of visual adaptation. The background color of the display screen was 0.5 gray level throughout the experiment.

### Electrophysiology

Both monkeys were chronically implanted in the left hemisphere over V1 with a microdrive system (Gray Matter Research, Bozeman, Montana, United States) which had 32 individually movable microelectrodes. Data acquisition was performed using the TDT system (Tucker-Davis Technologies, Alachua, Florida, United States). Signals were amplified and digitalized at 25 kHz (TDT PZ5 NeuroDigitizer). This raw signal was bandpass-filtered between 300 and 4000 Hz to extract multi-unit spiking activity (MUA), and low pass-filtered at 300 Hz and down-sampled with a decimation factor of 24 to about 1 kHz to retrieve the local field potential. MUA was isolated using the online detection algorithm in the OpenEx software (Tucker-Davis Technologies). Events crossing a threshold of 4 times the standard deviation of the filtered spiking band activity were considered as spikes and analyzed further.

### Data analysis

All decoding analyses were based on linear discriminant analysis (LDA). Firing rates across channels were treated as independent variables, i.e. predictors, with repeated measurements across trials. To test for stimulus-specificity, image identity was used as class label; to test for animal’s choice, match/nonmatch was used as class label. For time-resolved decoding, independent classifiers were trained at successive time points. In most decoding analyses, firing rate was calculated by binning spikes in moving causal windows of 150 ms and steps of 50 ms. For choice decoding, we used smaller step size (10 ms) to improve temporal resolution. Decoding accuracy for each session was measured by averaging the cross-validated classification performance over 20 repeated stratified sampling of the dataset (20 folds), and smoothed with cubic-spline functions to interpolate down to < 5 ms time resolution. Decoding accuracy values were averaged over sessions.

To test whether classifiers identified the animals’ choice rather than trivial differences in neuronal responses to match vs. nonmatch targets (c.f. Fig. 3), we trained classifiers on correct trials and tested the classifiers on incorrect trials. To this end, we took firing rate vectors at time point *t* = 2900 ms and trained decoders on correct trials (after excluding the trials with shorter reaction times) to predict match/nonmatch decisions. The accuracy for correct trials was obtained from 20-fold cross validation and averaging over sessions. In each cross-validation step, the decoders trained on correct trials were used to predict decision from incorrect trials, and the resulting decoding accuracy, averaged across sessions, was used as test performance on incorrect trials.

To identify subspaces containing stimulus and decision-related information, we used LDA to find low-dimensional projections (dimension of class number minus 1) of the data that optimized separation between classes and clustering within classes. We obtained the projection matrix for stimulus (2d) and decision (1d) subspaces at a particular time point (here *t* = 2700 ms) when both stimulus identity and the animal’s choice were readily decodable. We then projected firing rate vectors at each time point into these 2 subspaces using the aforementioned projection matrices, in order to evaluate the temporal evolution of the trajectories.

## Results

**Fig. 1:**
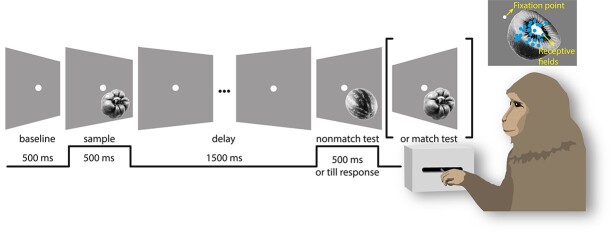
DMS paradigm. Inlet: positions of fixation point, stimulus, and receptive fields.

We trained 2 monkeys to perform a DMS task that required stimulus encoding, working memory, and a forced choice response (Fig. 1). During the task, the monkey fixated a spot at the centre of the screen. A sample stimulus was presented in the lower right visual field for 500 ms, which the animal had to remember over a delay of 1500 ms. After the delay a test stimulus was presented in the same location. The animal had to report whether the test stimulus was the same (match) or different (nonmatch) from the sample stimulus, by moving a mechanical lever forward or backward, respectively. The test stimulus was displayed for a maximum of 500 ms, or until the animal responded. Stimuli were standardized images (Brodeur et al. 2010; Brodeur et al. 2014) of simple objects displayed on a gray screen. A set of 3 stimuli were used in each session, and the stimuli varied between sessions. The size (4.4° and 7.84° of visual angle for Monkeys 1 and 2, respectively) and position (Monkey 1: 2.36° lateral of and 1.34° below the fixation spot; Monkey 2: 4.05° lateral of and 2.70° below the fixation spot) of the stimuli were tailored for each monkey to cover the ensemble of the receptive fields of the respective recording sites. Monkey 1 performed 6 sessions in total and 925.3 ± 181.8 (s.t.d.) trials per session (723.3 ± 147.9 correct trials; accuracy 78.1 ± 1.7% s.e.m., *n* = 6). Monkey 2 performed 10 sessions in total and 895.7 ± 210.5 (s.t.d.) trials per session (601.7 ± 157.8 correct trials; accuracy 66.8 ± 1.2% s.e.m., *n* = 10). Unless otherwise stated, only correct trials were analyzed. Data were analyzed separately for each animal; the results obtained from one animal are presented in the main text and the results of the second animal in the supplementary material.

**Fig. 2:**
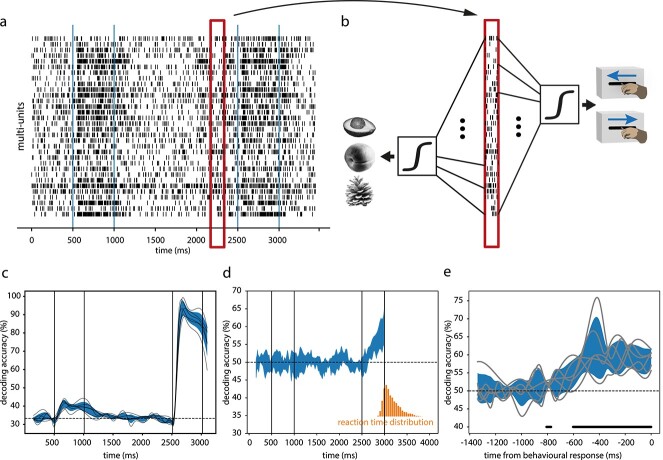
Decodability of stimulus identity and decision from neuronal population activity. (a) Raster plot of multi-unit activity in a single trial. The vertical lines mark sample and test stimulus time (same below). (b) Firing rate vectors in moving time window are used to train decoders to classify stimulus identity and behavioral choice. (c) Time-resolved accuracy of decoding test stimulus identity. (d) Time-resolved accuracy of decoding behavioral choice. The orange histogram shows the reaction time distribution. (e) Same as in (d) but trials were aligned to the time of behavioral response. The horizontal dotted lines denote chance level. The shaded areas indicate 95% confidence interval. The traces show results from individual sessions.

MUA was recorded from 32 recording sites in parallel with a chronically implanted microdrive system (Gray Matter Research, Bozeman, Montana, United States) while the animal performed the DMS task. To examine the amount of test stimulus-specific information contained in the response vectors, we trained decoders (linear discriminate analysis, LDA) at successive intervals (window size 150 ms, step size 50 ms) to predict test stimulus identity (Fig. 2a-b). The decoding accuracy (interpolated at finer timescale) increased quickly after response onset, peaked at about 2692 ms, and maintained a high level as long as the stimulus was present (Fig. 2c for Monkey 1. Supplementary fig. 1b for Monkey 2). For example, at 2700 ms, which is 200 ms after test stimulus onset, decoding accuracy for the test stimulus was 93.25 ± 1.46% (s.e.m.), significantly above chance level (33.3% for 3 stimuli; *t* = 37.36, *P* < 0.01, *n* = 6; one sample *t*-test).

To investigate whether the activity of V1 neurons contained information about the forthcoming choice (match/nonmatch), we trained decoders on firing rate vectors to predict whether the animal would move the lever forward or backward. The decoding accuracy for the forthcoming choice initially crossed the chance level (50% for 2 choices) at 157 ms after test stimulus onset, and increased monotonically afterwards (Fig. 2d for Monkey 1; *t* = 3.14, *P* = 0.0039, *n* = 6, at 2700 ms. Supplementary Fig. 1c for Monkey 2). The average reaction time was 632.0 ± 174.7 ms (s.t.d.) for Monkey 1 and 503.1 ± 201.5 ms for Monkey 2. Thus, it was possible to decode the monkey’s forthcoming choice well before the monkey responded. Aligning the trials to response times revealed that the forthcoming choice was decodable already 500 ms before response onset (Fig. 2e for Monkey 1; *t* = 12.23, *P* < 0.01, *n* = 6. Supplementary Fig. 1d for Monkey 2). Even though the firing rates of responses to a given test stimulus were identical irrespective of whether the stimuli were matching or nonmatching (Supplementary Fig. 2), the decoders may have picked up some differences in the responses to matching and nonmatching stimuli (stimulus type-related cues) rather than decision-related cues. In order to exclude the former we trained decoders to distinguish between match and nonmatch outcome in correct trials and tested the decoders in error trials, in which the relationship between test stimulus type and the animal’s choice was inversed. In this case the performance of the decoders was significantly below chance (Supplementary Fig. 3; 43.69 ± 1.64%, *t* = −3.84, *P* = 0.012, *n* = 6 for Monkey 1, 46.12 ± 1.24%, *t* = −3.14, *P* = 0.012, *n* = 10 for Monkey 2), indicating that the decoders systematically misidentified match vs. nonmatch test stimulus types while predicting correctly the forthcoming choice.

**Fig. 3:**
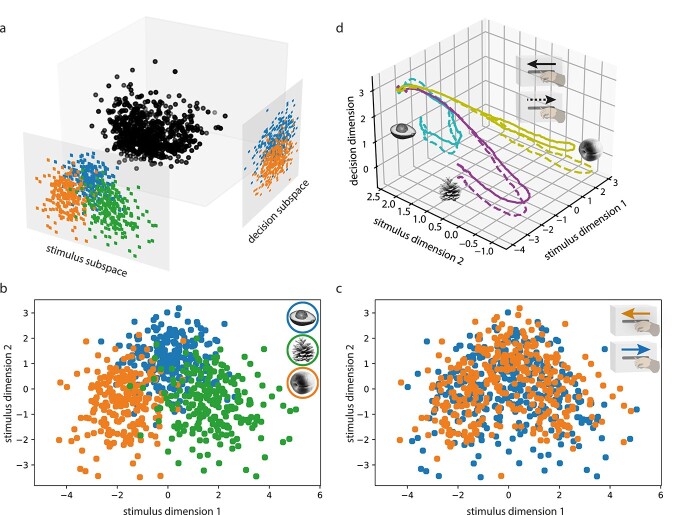
High-dimensional coding of stimulus and decision information. (a) Schematic representation of the joint presence of stimulus and decision information in 2 subspaces. (b) Projection of data in the stimulus subspace, where points were colored based on stimulus identity. (c) Same projection as in (b), but points were colored based on match/nonmatch decision. (d) Projection of test stimulus-evoked firing rate trajectories into the 3D space spanned by stimulus and decision axes. The colors indicate test stimulus identity. The solid and dashed lines denote conditions of moving lever forward and backward, respectively.

These results indicate that the responses to the test stimulus contain information on both stimulus identity and the pending decision, raising the question how this information is encoded. Since population activity is high-dimensional, various codes could coexist in subspaces without interference (Fig. 3a). To test this possibility, we examined the subspaces obtained from the previous decoding analysis using LDA. LDA performs simultaneously a supervised classification and dimensionality reduction (linear subspace identification) by finding the low-dimensional projection of data that maximizes separation between classes and minimizes dispersion within classes. This optimized projection thus represents the subspace that is most informative for discriminating different classes. Here, we used the firing rate vectors at a particular time point (*t* = 2700 ms) in the trial, when both stimulus and choice-related information was decodable, to obtain an optimal 2-dimensional stimulus space and a 1-dimensional decision subspace. In the stimulus subspace, there was clear clustering of data points according to stimulus identity (Fig. 3b), but complete overlap of the match/nonmatch decision conditions (Fig. 3c). To obtain an intuition about the dynamics of population activity, we projected the neural activity over time (from test stimulus onset) into the space spanned by the stimulus subspace and decision subspace. The trajectories of population activity separated for the 3 stimuli, but were also systematically deflected along the decision axis (Fig. 3d for Monkey 1 and Supplementary fig. 4 for Monkey 2).

## Discussion

The results of this study show that the responses of the same neuronal population in monkey V1 can simultaneously represent information about the identity of visual stimuli and about the forthcoming choice of a particular motor response. The stimulus-specific and decision-related information existed without apparent overlap in different subspaces of the high-dimensional population activity.

The finding that choices can be decoded from V1 responses agrees with previous studies reporting choice-related signals in primary sensory areas (Nienborg and Cumming 2009, 2014; Siegel et al. 2015; Kwon et al. 2016) and it also agrees with the evidence from large-scale recordings that responses associated with sensory stimulation, behavioral choice, and motor actions are widely distributed across multiple brain areas (Pinto et al. 2019; Steinmetz et al. 2019). The choice-related information is with all likelihood not generated within V1 but conveyed by projections from higher cortical areas (Markov et al. 2013; Markov and Kennedy 2013). In rodents performing a sensory discrimination task, choice-related activity first emerges in premotor and motor cortical areas before spreading to primary sensory cortex (Orsolic et al. 2021; Zatka et al. 2021). Accordingly, precisely timed optogenetic inactivation of sensory cortex after stimulus encoding does not impair the animals’ discrimination performance (Guo et al. 2014; Zatka et al. 2021), supporting the notion that choice-related information originated from higher order areas. Therefore, decodability of choice-related information from V1 or any other cortical area does not imply that the information is actually encoded in the same way by the brain, nor does it imply that this decodable information is used by the brain, although it can probably be exploited when required.

The coexistence of decision- and stimulus-related signals in V1 raises the question how these different types of information are encoded in the activity of the same population of neurons. In the prefrontal cortex neurons typically exhibit mixed selectivity and respond to heterogeneous task parameters (Rigotti et al. 2013). This mixed selectivity allows for the flexible association of task-related information and is considered a hallmark of distributed coding in the high-dimensional dynamic space exploited by computations in recurrent networks (Rigotti et al. 2013; Singer and Lazar 2016; Parthasarathy et al. 2017). Our analyses showed that the stimulus- and choice-related information is encoded in different subspaces spanned by the activity of the same neural population (Mante et al. 2013; Elsayed et al. 2016; Rademaker et al. 2019; Aoi et al. 2020; Libby and Buschman 2021), and that these subspaces are likely orthogonal to each other (Semedo et al. 2019; Perich et al. 2020). This permits superposition of stimulus-specific information or different motor programs in the same network without leading to catastrophic interference (Zeng et al. 2019). Studies on fading memory, a hallmark of the dynamics of recurrent networks have shown that such superposition is possible in the V1 (Nikolić et al. 2009). In the present experiment the subspace initially filled with stimulus information was subsequently complemented with another subspace occupied with the choice-related signal, supporting the notion that subspaces can be flexibly deployed depending on task demand.

These results raise the question, why decision- or choice-related information should be back-propagated to V1 (Singer and Lazar 2016). One interpretation is that top-down signals reset the dynamics in V1 once the perceptual discrimination task is accomplished in order to prepare V1 for the processing of novel input. However, this would not require to inform V1 about the outcome of the perceptual process, match or nonmatch. A more likely interpretation is suggested by the complex recurrent interactions among cortical areas that result from abundant reciprocal coupling (van den Heuvel and Sporns 2011; Markov et al. 2013; Markov et al. 2014). Recurrently coupled networks process signals in a highly parallel manner and represent computational results in complex dynamical landscapes to which all nodes of the network contribute continuously. Any local signal spreads immediately over the whole network (Effenberger et al. 2022). One out of many signatures of these dynamics are traveling waves that are considered hall marks of cortical networks (Muller et al. 2018; Keller and Welling 2023).

Thus, we propose that any cortical area involved in the present experiment comes to represent the various results of the distributed computations as soon as these become available. Initially, the only information available is provided by the retina. After comparison with information stored in working memory, the match/nonmatch information becomes available and spreads across the interconnected areas. In V1 this information must fill a dynamic subspace that is separate from the subspace representing stimulus-related information in order to avoid interference. In this scenario, the different delays with which the dynamic coding space of V1 is filled with retinal and later with choice-related activity do not reflect the conduction delays of a serial process that relays signals from V1 all the way up to the decision-making areas and then propagates the result back to V1 in reverse order across the processing hierarchy. Rather, the sequential filling of the representational space would reflect the sequential convergence of the whole interconnected network towards the solution of the task. Support for this interpretation comes from considerations of conduction delays. These are in the range of maximally a few tens of milliseconds, c.f. synfire chains (Abeles et al. 1993; Ikegaya et al. 2004) and thus negligibly short compared with processing times. A testable prediction of this scenario is that choice-related signals should become available at about the same time, allowing for a few milliseconds for conduction delays, in all interconnected areas. This parallelized processing strategy is faster than serial processing and allows task-related information to be simultaneously available in various dynamic subspaces of the engaged processing area.

## Supplementary Material

supplementary_materials_bhad420

## Data Availability

The data used in this study will be made available upon request.

## CRediT author statement

Yiling Yang (Conceptualization, Data curation, Formal analysis, Investigation, Methodology, Project administration, Software, Visualization, Writing—original draft, Writing—review & editing), Johanna Klon-Lipok (Investigation, Methodology, Project administration, Resources), Wolf Singer (Conceptualization, Funding acquisition, Investigation, Methodology, Project administration, Resources, Supervision, Writing—review & editing).
