# Supplementary material for: Joint encoding of stimulus and decision in monkey primary visual cortex
Source: Cereb Cortex. 2023 Nov 11;34(1):bhad420. doi: 10.1093/cercor/bhad420 (PMC10793581; doi:10.1093/cercor/bhad420)
Supplement: supplementary_materials_bhad420 [file supplementary_materials_bhad420.docx]

Joint Encoding of Stimulus and Decision in Monkey Primary Visual Cortex

**Supplementary Materials**

Yang Yiling^1^, Johanna Klon-Lipok^2^, Wolf Singer^1,2,3^*

^1^Ernst Strüngmann Institute (ESI) for Neuroscience in Cooperation with Max Planck Society, Deutschordenstraße 46, 60528 Frankfurt am Main, Germany

^2^Max Planck Institute for Brain Research, Max-von-Laue-Str. 4, 60438 Frankfurt am Main, Germany

^3^Frankfurt Institute for Advanced Studies, Ruth-Moufang-Str. 1, 60438 Frankfurt am Main, Germany

*Corresponding author.


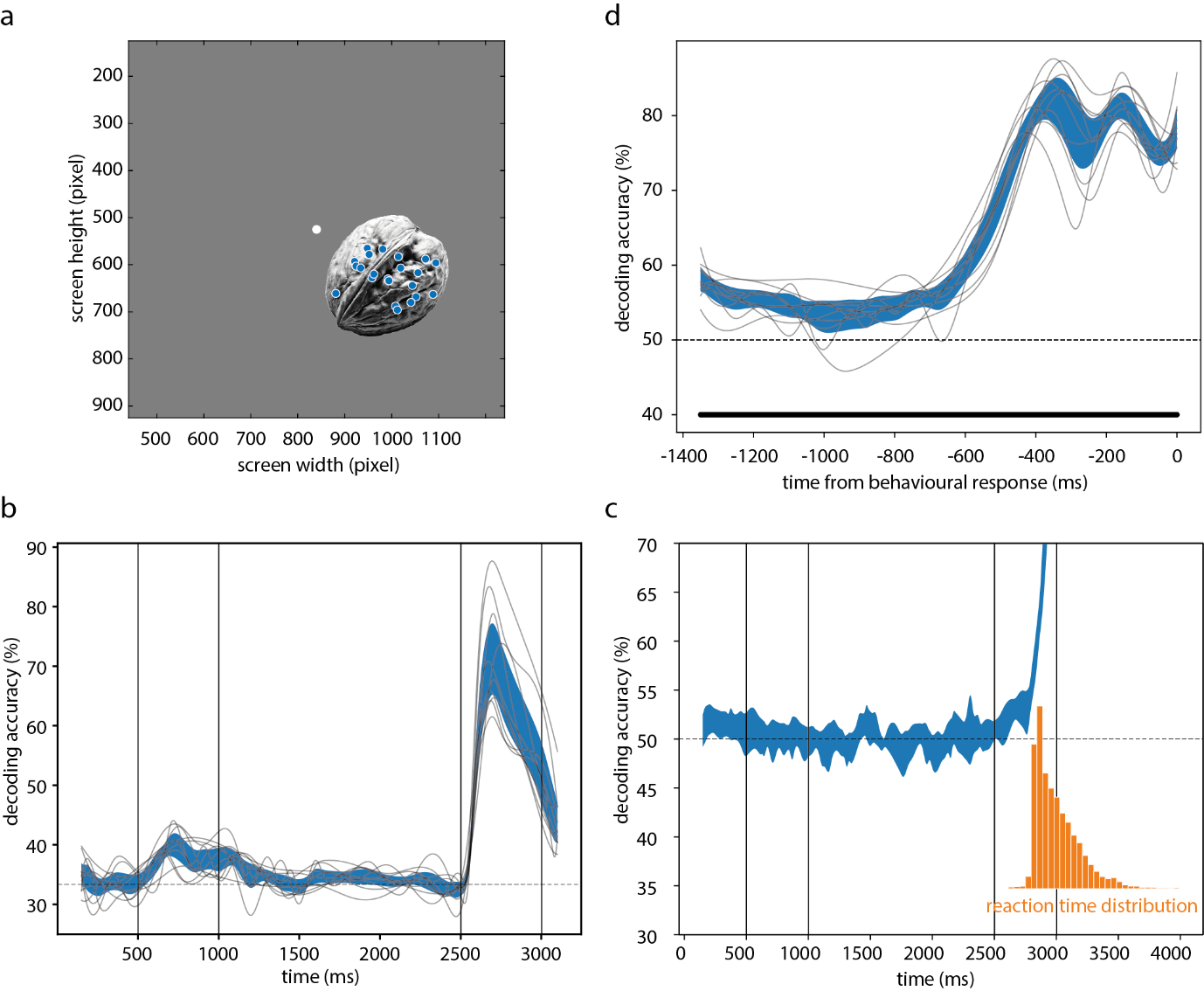


Supplementary figure 1 Decodability of stimulus identity and decision from neuronal population activity (Monkey 2). (a) Positions of fixation point (white), stimulus and receptive fields (blue). (b-d) Same convention as Figure 2 c-e. (b) Time-resolved accuracy of decoding test stimulus identity. (c) Time-resolved accuracy of decoding behavioural choice. Orange histogram shows the reaction time distribution. (d) Same as in (c) but trials were aligned to the time of behavioural response. Blue shaded areas indicate 95% confidence intervals (n = 10). Grey traces show results from individual sessions.


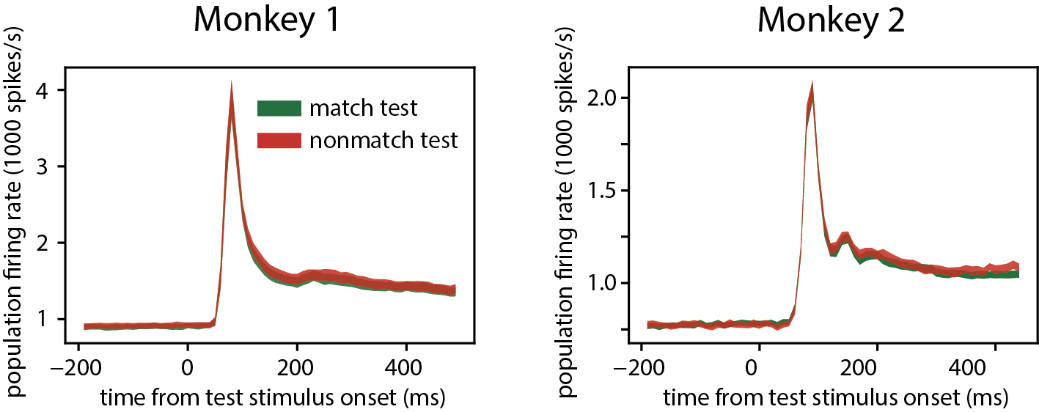


Supplementary figure 2 Population firing rate responses to test stimuli in match vs. nonmatch trials. Population firing rates are summed across channels (multi-units) and averaged across trials. Error shades indicate 95% confidence intervals.


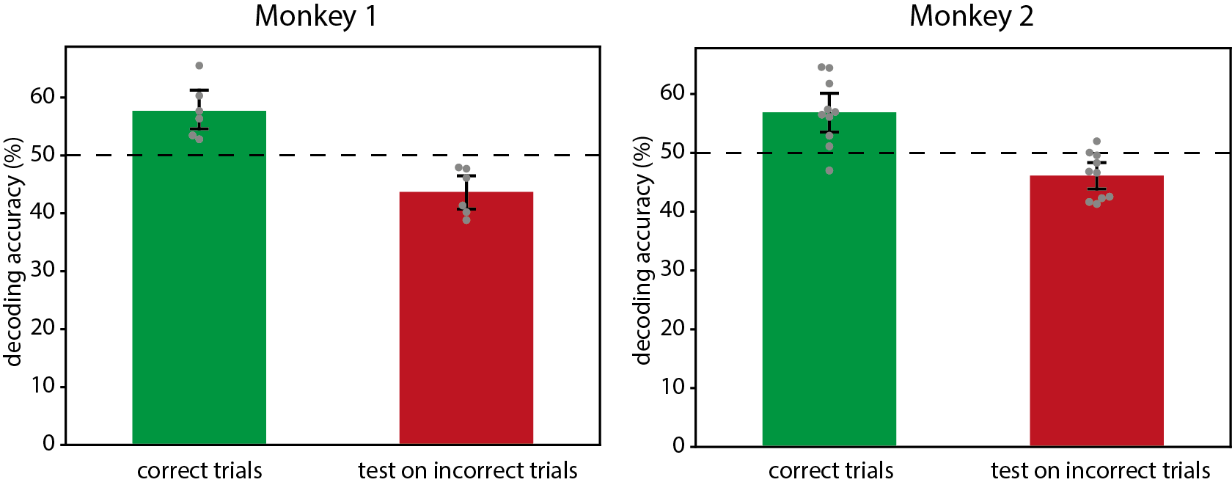


Supplementary figure 3 Decoding accuracy for match/nonmatch decision on correct trials and test performance on incorrect trials. Error bars denote 95% confidence intervals. Cross-validated decoding accuracy on correct trials: 57.58 ± 1.93%, t = 3.98, p = 0.011, n = 6 sessions, Monkey 1; 56.88 ± 1.79%, t = 3.85, p = 0.0039, n = 10 sessions, Monkey 2. Test accuracy on incorrect trials using classifiers trained on correct trials: 43.69 ± 1.64%, t = −3.84, p = 0.012, Monkey 1; 46.12 ± 1.24%, t = −3.14, p = 0.012, Monkey 2.


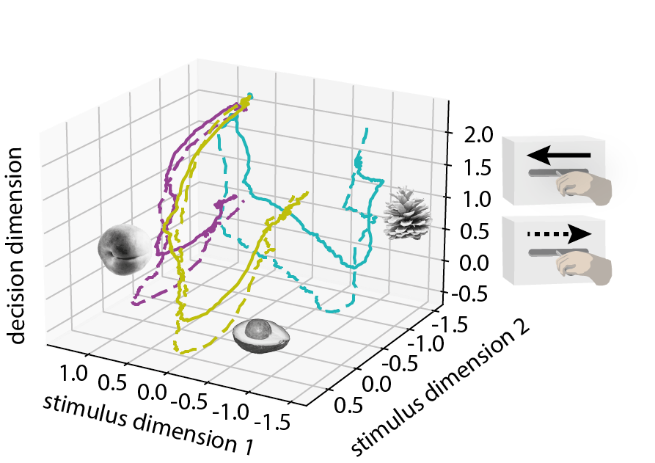


Supplementary figure 4 High-dimensional coding of stimulus and decision information (Monkey 2). Same convention as in Figure 3d. Projection of test stimulus-evoked firing rate trajectories into the 3D space spanned by stimulus and decision axes. Colours indicate test stimulus identity. Solid and dashed lines denote conditions of moving lever forward and backward, respectively.
